# The Intriguing History of Cancer Immunotherapy

**DOI:** 10.3389/fimmu.2019.02965

**Published:** 2019-12-17

**Authors:** Paula Dobosz, Tomasz Dzieciątkowski

**Affiliations:** ^1^Department of Hematology, Oncology and Internal Medicine, Medical University of Warsaw, Warsaw, Poland; ^2^Chair and Department of Medical Microbiology, Medical University of Warsaw, Warsaw, Poland

**Keywords:** history of immunotherapy, immunotherapy, cancer immunotherapy, CAR T, oncolytic virus, checkpoint inhibitors

## Abstract

Immunotherapy is often perceived as a relatively recent advance. In reality, however, one should be looking for the beginnings of cancer immunotherapy under different names as far as in the Antiquity. The first scientific attempts to modulate patients' immune systems to cure cancer can be attributed to two German physicians, Fehleisen and Busch, who independently noticed significant tumor regression after erysipelas infection. The next significant advances came from William Bradley Coley who is known today as the Father of Immunotherapy. It was Coley who first attempted to harness the immune system for treating bone cancer in 1891. His achievements were largely unnoticed for over fifty years, and several seminal discoveries in the field of Immunology, such as the existence of T cells and their crucial role in immunity in 1967, stepped up the research toward cancer immunotherapy known today. The following paper tracks cancer immunotherapy from its known beginnings up until recent events, including the 2018 Nobel Prize award to James Allison and Tasuku Honjo for their meticulous work on checkpoint molecules as potential therapeutic targets. That work has led to the successful development of new checkpoint inhibitors, CAR T-cells and oncolytic viruses and the pace of such advances brings the highest hope for the future of cancer treatment.

## Introduction

We tend to think that immunotherapy is a very recent medical achievement, originating no later than a couple of decades ago. As a matter of fact, the very beginning of immunotherapy *sensu lato* might be traced back to the China's Qin dynasty period, around the third century BC (1). Although difficult to prove, scarce written resources mention purposeful inoculation with variola minor virus in order to prevent smallpox disease (1, 2). Many centuries later, in 1718, this practice was also reported in the Ottoman Empire by Lady Mary Wortley Montague, the wife of the British ambassador residing in Istanbul (1). Inspired by local custom and its positive outcome, she tried to popularize inoculation on her return to England but met with no success due to the resistance and general disbelief of British physicians (1). Nevertheless, in 1765, Dr. John Fewster presented a similar report in front of the London Medical Society members (1). Not long after that, in 1796, Edward Jenner demonstrated protective immunity against smallpox through inoculation with common cowpox virus (1). This event was largely accepted as the beginning of the vaccinations era which undoubtedly transformed modern medicine and saved millions of lives worldwide.

**Figure 1:**
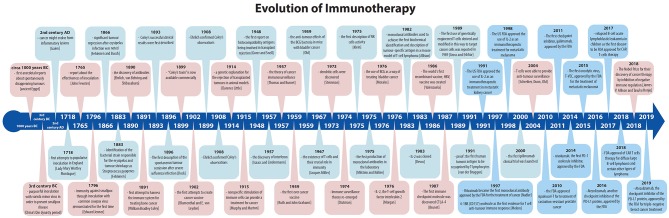
The history of immunotherapy; major breakthroughs have been indicated, including FDA approvals related to the field.

The history of vaccinations, no matter how appealing and wonderful, will not be described in detail in this paper. Instead, we will track the relatively modern part of the history of immunotherapy, immunotherapy *sensu stricte*, focusing on cancer treatment from the very first attempts up to the 2018 Nobel Prize winners James P. Allison and Tasuku Honjo for their discovery of cancer therapy by inhibition of negative immune regulation (Figure 1).

## The Beginnings

From ancient Egypt, some 3,000 years ago, to the early nineteenth century there have been multiple anecdotal reports of tumors disappearing spontaneously or after an infection with concomitant high fever (3, 4). The similarity between cancer and inflammation was described for the first time by the Greek physician, Galen, who noted that cancer might evolve from inflammatory lesions (5). The first scientific attempts to modulate patients' immune systems to cure cancer can be attributed to two German physicians, Fehleisen and Busch, who independently noticed significant tumor regression after erysipelas infection (4). They both described their observations and tried to repeat them later on, with little success (4). Eventually, Fehleisen managed to properly identify the bacterial strain responsible for the erysipelas and tumor shrinkage as *Streptococcus pyogenes* (4). The next significant advances came from William Bradley Coley who is known today as the Father of Immunotherapy. Coley first attempted to harness the immune system for treating bone cancer in 1891 (6, 7). He directly observed a number of cases in which cancer patients went into spontaneous remission after developing erysipelas—a streptococcal skin infection (7). He also delved into medical records, epicrisis and medical literature accessible to him at the end of nineteenth century, including the works of his predecessors, and discovered as many as 47 case reports of patients with potentially incurable cancers which underwent spontaneous remission after concomitant acute bacterial infection (1, 4). Spontaneous tumor regression is extremely rare, occurring in ~1 in 60,000–100,000 cancer patients worldwide. It is, however, a widely accepted phenomenon with case reports being regularly published worldwide in contemporary medical journals (4). From 1891 Coley took things a step further; he began injecting different mixtures of live and inactivated *Streptococcus pyogenes* and *Serratia marcescens* into patients' tumors and thus could be said to have developed the first immune-based treatment for cancer (1, 6, 7).

Although his successful clinical results were first described in May 1893, Coley was not esteemed in the medical society (1, 8). He achieved durable and complete remission in several types of malignancies, starting from sarcoma, lymphoma, and testicular carcinoma and reported over 1,000 regressions or completely cured patients (4, 6, 7). Despite this success, the lack of a known mechanism of action for the “*Coley's toxins*” (available commercially from 1899) as well as the risks of infecting cancer patients with highly pathogenic bacteria, caused oncologists to prefer surgery and radiotherapy in the early twentieth century (1, 6, 8, 9). Coley's legacy was consequently widely forgotten and even denied for some decades.

Interest in the immune system burst again after 1945, with many advances in immunity and cancer research such as the discovery of interferon (10) or the very successful work of Ruth and John Grahams on the first ever cancer vaccine. The latters' work was largely unnoticed despite 22% of patients involved in the trial having stable disease or cancer remission (1). The existence of T cells and their crucial role in immunity was not obvious until 1967, when Jacques Miller characterized their function in his pivotal “*Nature*” publication (11). Six years later dendritic cells were discovered (1973, Steinman) closely followed by the first description of natural killer cell (NK cells) activity (1975, Klein) (1, 12–14). In the meantime, accumulating knowledge of immunology allowed researchers and physicians from the University of Minnesota to pioneer bone marrow transplant as a treatment for hematological cancers, a method that is still used today (15). The early trials of transplantation as a method of cancer treatment were tested for over a centurybefore that first success, mostly on mice. Efforts were intensified after Clarence Little presented a genetic explanation for the rejection of transplanted tumors in animal models in 1914 and further boosted in 1948 after the first report on histocompatibility antigens being involved in transplant rejection (4, 16).

Finally, in the 1980's, when the first vaccine based on a single cell surface antigen became available in a form of hepatitis B vaccine, the field of immunotherapy ultimately re-emerged (1). Optimism resurfaced that immunotherapy might be used to treat many diseases, including cancer, and propelled research into where we are at the moment.

## Immune System in Charge

Nearly fifty years ago professor Lloyd J. Old, pioneer of cancer immuno-oncology, noted that “there is something unique about a cancer cell that distinguishes it from normal cells, and that this difference can be recognized by the body's immune system” (17). He correctly predicted that in the future immunotherapy would be a fourth kind of cancer therapy, together with surgery, chemotherapy, and radiotherapy but he expected much faster progress (17). Several decades of intensive research and clinical trials passed before cancer immunotherapy reached its legitimate place as a fourth pillar of cancer treatment.

In 1908 Paul Ehrlich confirmed Coley's observations reporting several tumors being spontaneously suppressed by the actions of the immune system (8). At the same time Murphy and Morton of the American Rockefeller Institute were conducting mice experiments which led them to formulate their 1915 hypothesis that even nonspecific stimulation of immune cells, particularly lymphocytes, can provide a treatment for cancer (18). Human trials over the following years were, however, very unsuccessful and led to hypothesis dereliction and dismissal of the entire idea of cancer immunotherapy for decades (8).

The strategy of using bacteria to treat cancer emerged again in 1976 when a trial was conducted to examine the use of the Bacille Calmette-Guérin (BCG), the tuberculosis vaccine, as a way of preventing the recurrence of bladder cancer (4, 6, 19). The idea came from a 1959 study conducted by Old and his team, demonstrating the anti-tumor effects of the BCG bacteria in mice with bladder cancer (4, 20). BCG vaccine is made of weakened, live bacteria related closely to those causing tuberculosis (21). They were injected in solution into the bladder of cancer patients and left there for several hours in order that the bacteria might trigger the patients' immune response (21, 22). This is an example of a very successful cancer therapy involving activated macrophages in tumor rejection (22). BCG therapy has been shown to be very effective and it continues to be used today in non-muscle invasive bladder cancer (4, 6). It is also a therapeutic confirmation of Coley's original principles.

Bacterial infections were not the only focus for researchers working on potential triggers of immune response against tumors. After the discovery of the virus at the turn of the nineteenth century interest focused on viral diseases. The first spontaneous tumor remission was documented by the American physician George Dock in 1896 after a woman with leukemia went through a cancer remission after severe influenza infection (23). Despite this it was not until the beginning of the twentieth century that viruses and viral diseases could be connected with cancer through academic investigation.

The next milestone in cancer immunotherapy was when Thomas and Burnet first proposed their excellent theory of cancer immunosurveillance (6). It was 1957 when they first suggested that lymphocytes might act as sentinels in order to identify and possibly eliminate somatic cells transformed by mutations (6, 24). Again, the lack of data and understanding of the mechanisms of tumor-specific antigens, as well as the technical inability to grow lymphocytes *in vitro*, postponed any further progress for many years (6). Immune surveillance theory re-emerged in 1974, when Stutman showed that nude mice with impaired immune system functions develop cancer more readily than wild type strain (25–27). About the same time natural killer cells were identified, providing additional support for the power of human immune system (8, 28, 29). Burnet and Thomas described their elegant hypothesis of cancer immunosurveillance in the mid- twentieth century, but it was not until the end of the twentieth century that Schreiber, Dunn, Old and their teams proved that T cells were able to provide anti-tumor surveillance and anti-tumor immune responses (1, 24, 30–32). Further discoveries followed including mechanisms for immunoediting, evidence for cancer cell escape and the recognition that immunosuppressed patients have significantly higher risk of cancer development (4, 31, 33).

In 1991 van der Bruggen and colleagues identified the first human tumor antigen to be recognized by T lymphocytes (34). Later they provided the first identification of a real molecular target through cloning the melanoma antigen encoding gene (MAGE), a gene encoding an antigen recognized by the cytotoxic T cells (34).

## Antibody-Based Therapies

The discovery of antibodies around 1890 has been variously attributed to Paul Ehrlich, Emil von Behring and Kitasato Shibasaburo and the small proteins have since become well-established forms of treatment in a wide spectrum of diseases, including cancer (35, 36). They act in a several ways, for example by preventing an antigen from attaching to its receptor on the cell's surface or by marking an antigen to be destructed (21). Monoclonal antibodies are usually used, “mono” inferring they are a single type of an antibody, targeting a specific antigen and “clonal” implying they are multiplied thousands of times in order to gain a therapeutic, clinically effective dose (21).

The principle antibody action is to attach to the cell's antigens and mark the cancer cell to be destroyed by specialized immune system cells (21). Some antibodies work by signaling the immune system and triggering it to carry on the attack, whereas other antibodies may interrupt the signaling which tells cancer cells to grow, divide and spread (21). Milstein and Köhler pioneered the production of monoclonal antibodies in the laboratory in the 1970s (6). They used so called “hybridomas,” antibody-secreting cell lines made by the fusion of lymphocytes and myeloma cell lines (6, 37). Research on antibody-based therapies bloomed during the following decades and eventually led to the development of rituximab, a monoclonal antibody which binds to CD20 protein present on the surface of immature B cells (6). In 1997 Rituximab became the first monoclonal antibody approved by the FDA for the treatment of cancer, non-Hodgkin's lymphoma (6, 38). The drug targets immature B cells for elimination by the NK cells (6, 38).

Another important molecule worth mentioning is 4-1BB (CD137), discovered in the late 80's on the surface of activated cells, thus initially named “induced lymphocyte activation” (ILA) molecule in humans (39). As a member of the tumor necrosis factor receptor superfamily, this glycoprotein binds to its ligand (4-1BBL, also known as CD137L) expressed on several cell types, including antigen-presenting cells, activated B cells, macrophages and some tumor cells too (39, 40). Observations made by Melero et al. in 1997 suggested a significant role of this molecule in the amplification of the T cell mediated immune response, and further experiments involving mAbs not only showed its potent role in anti-cancer therapy, but also reasserted the evidence of the T cell mediated immune response as such (39–41). Further studies supported the therapeutic potential of targeting the pathway involving 4-1BB molecule in cancer treatment, resulting in many currently ongoing clinical trials (39).

It would be incorrect not to mention trastuzumab (Herceptin), a well-known monoclonal antibody that attaches itself to the growth factor antigen present on certain types of breast cancer cells, stopping those cells from growing and dividing and leading inevitably to their death (21).Some monoclonal antibodies are called “conjugated antibodies” because they are attached to another chemical or radioactive agent (21). This chemical modification is helpful in localizing cancer cells and/or destroying them.

The most promising antibodies currently tested in cancer research are checkpoint inhibitors, with several drugs already approved by the FDA for more than nine cancer types (42). In 1982 James Allison and colleagues used monoclonal antibodies to achieve the first biochemical identification and description of tumor-specific antigen in a mouse model of T-cell lymphoma (43). Just one year later they identified the first T cell antigen receptor (44). The immunotherapy era has re-emerged, this time successfully. In 2000, the first ipilimumab clinical trial was launched, starting an avalanche of similar studies that continue up to this day. The notion of immune checkpoint blockade has transformed the entire field today and saved thousands of lives (42). As an indication of the success of this targeted approach the FDA has approved one drug for every tumor possessing a particular genetic makeup, an advance from traditional tissue-of-origin cancer approaches to therapeutic classification (42). The first immune checkpoint molecule was discovered in 1987 and named cytotoxic T-lymphocyte antigen number 4 (CTLA-4) by Brunet and his team (45). However, the function of this molecule remained ambiguous until 1995, when Jim Allison et al. pinpointed it as a crucial immune checkpoint molecule with great potential as a future anti-cancer therapy target (46, 47).

The first CTLA-4 blocking antibody was immediately developed and tested on animals a year later, in 1996 (47). The first checkpoint inhibitor approved by the FDA was ipilimumab in 2011 for the therapy of advanced melanoma (1, 42). Today it is approved for several cancer types and the most promising result is that over 20% of the patients enrolled in the first ipilimumab clinical trials (before the 2011 approval) are still alive and show no evidence of disease (4, 42).

Another checkpoint inhibitor, nivolumab, followed in 2014 and was the first PD-1 molecule inhibitor approved by the FDA (42). In 2014 Nivolumab became the first PD-1 inhibitor to gain regulatory approval for the treatment of melanoma in Japan (6). In the next 4 years several other inhibitors of the PD-1 receptor or its ligands, PD-L1 and PD-L2, were approved worldwide with pembrolizumab, atezolizumab, durvalumab, and avelumab, showing significant improvement in several cancer types (42). Atezolizumab, formerly known as MPDL3280A, is another checkpoint inhibitor of the PD-L1 protein, approved from 2016 for the treatment of melanoma, lung cancer, bladder cancer, as well as triple-negative breast cancer treatment from March 2019 (6, 48–50).

All of the above immune checkpoint molecules, including cytotoxic T lymphocyte antigen 4 (CTLA-4) or programmed cell death-1 (PD-1) and its ligands (PD-L1 and PD-L2) are known to be expressed on tumor-infiltrating lymphocytes (TILs) as well as some tumor cells (6). When expressed on the tumor site they allow cancer cells to evade immune responses so immune checkpoint blockage was a crucial breakthrough in cancer treatment (6). With prior conventional treatments only about 4% of patients enrolled in the clinical trials of the above drugs would be expected to be alive today. Thanks to the received antibodies this percentage is much higher, with between 16 and 30% (and in some reports as high as 50%) of melanoma and lung cancer trial patients surviving to date (4, 42).

## IL-2, Interferons and Other Cytokines

Cytokines are small proteins naturally produced and secreted by several immune system cells. They are crucial in signaling between immune cells, as well as between immune cells and several other cell types in the body (21). The first cytokine to be discovered was interferon alpha, also known as type I, described in 1957 Isaacs and Lindenmann (10). IL-2, the T-cell growth factor interleukin 2, was identified in 1976 (51) and allowed investigators to culture lymphocytes T *in vitro* for the very first time (6). IL-2 was cloned in 1983 and was immediately harnessed in clinical trials leading to promising results including tumor shrinkage (52–54). It proved to be effective if administered in large quantities to patients with metastatic cancers through enhancing the production of lymphocytes T. It is thus usually called “immunostimulatory cytokine”) (4, 6, 55). The US FDA approved the use of interleukin 2 as an immunotherapeutic treatment in 1991 for the treatment of metastatic kidney cancer and in 1998 for metastatic melanoma (6, 56).

## Immunosuppression-Reducing Treatments

Cancer immunotherapy is changing cancer treatment paradigms, but response rates to several existing treatment types remain low. This at least partially can be explained by the lack of host's pre-existing anti-tumor immunity (57, 58). Moreover, one of the cancer hallmarks is the avoidance of the immune system's potential attack, the escape from the immune control, and remain invisible to the immune cells (57). It is important to remember that tumor is composed of cancer cells, but also stromal features, such as fibroblasts, blood vessels and infiltrating immune cells among others (57). All those elements are collectively named tumor microenvironment (TME) and remain of utmost importance for the immunotherapy success (57, 58). Tumor intrinsic immunosuppressive features can also inhibit effector T cell function, especially regions of hypoxia or elevated lactate levels in the TME (57, 59). In fact, TME is highly variable between individuals and different tumors themselves, therefore many preclinical and clinical trials are targeting novel targets related to the TME, especially the TME-mediated immunosuppressive pathways (57, 58, 60). Among those pathways the most extensively investigated are: downregulation of the MHC class I on the surface of tumor cells (in order to avoid detection by the CD8+ effector cells), downregulation of the FAS and/or TRAIL molecules (in order to avoid tumor cell killing), as well as targeting crucial enzymes (such as enzyme indoleamine 2,3-dioxygenase, IDO) or several cytokines, such as VEGF, TGFβ or IL-10 (57, 61, 62). There are also ongoing studies investigating therapeutic approaches targeting immune mediators (such as legumain), cytochromes (for example CYP450), or suggesting the use of nanotechnology to erase existing TME suppressive influence (63–65).

Most of the drugs used in the cancer chemotherapy have immunosuppressive effect (66, 67). Moreover, it has been noticed nearly 50 years ago that some patients have manifested new tumors in different locations while their original neoplasm have been treated (66). Furthermore, increased incidence of cancer is observed in immunosuppressed patients, what at least partially had been attributed to the actions of Tregs in 1995 (57, 68). Of all the tumor infiltrating cells, regulatory T cells (Tregs) play a crucial role in moderating immune destruction, promoting immunosuppression by several ways, especially secretion of immunosuppressive cytokines (57, 68, 69). Other cells present in the TME, such as myeloid-derived suppressor cells (MDSCs) tumor-associated macrophages (TAMs) and mast cells are usually upregulated in the TME, preventing the immune system from eliminating tumor cells (64, 67, 69).

Finally, the presence of tumor-associated macrophages, especially type M2, inside the TME has been associated with poor prognosis (57, 70). Being the most abundant cells infiltrating human tumors, they are capable of suppressing immune responses (70). Therefore, several therapies targeting tumor-infiltrating macrophages have been recently invented, for example depleting macrophages count with anti-colony stimulating factor 1 antibodies (71).

## Cancer Vaccines

There is probably no medical innovation which has had a more significant impact upon medicine and global health than the invention and development of vaccinations. Just as our immune system works unceasingly to prevent infections, protecting us from potentially harmful bacteria, viruses and parasites, the immune system also plays a pivotal role in cancer prevention (21). It is possible to enhance this function either by preventing infection or by “teaching” immune system cells to recognize and kill cancer cells once they arise in the body. Several FDA-approved cancer prevention vaccines have been in use for the past two decades. These include the hepatitis B (HBV) vaccine and the human papillomavirus (HPV) vaccine, both of which prevent infection by cancer-causing viruses (21, 72). The impact of viral carcinogenesis is becoming increasingly evident and prevention through vaccination is the most important and effective way of lowering such cancer incidence.

Apart from the preventative role of vaccines such as HPV and HBV vaccines, there is also intensive, on-going research on vaccines targeting existing cancer, the goal of cancer immunogeneticists and immunooncologists for a long time. Perhaps a therapeutic cancer vaccine could be used to treat cancer which has already emerged? It is well-known that some cancer cells can evade immune cells or even suppress their activity and linger unnoticed for many years in the body (22). Many types of cancer cells can express specific ligands for immunosuppressive checkpoint proteins on their surface, thus preventing the immune system from attacking the growing tumor (22). Later they start dividing and spreading unchecked, leading to the tissue damage, tumor formation and eventually death (21, 73).

There are two main types of therapeutic cancer vaccines, autologous and allogenic cancer vaccines (21). The first type is a personalized cancer vaccine made from a patient's own cells, based either on cancer cells or immune system cells. The cells are taken from the individual, processed and multiplied in the laboratory and then reinjected into the patient's circulation. The processed cells recognize cancerous cells and trigger the immune response against the cancer (21). This type of treatment would be used together with other cancer therapies, such as surgery or radiation, in order to eradicate trace amounts of persisting cancer cells. Ideally, some memory cells would remain in the patient's system with the promise that they might respond immediately if cancer cells appear again (21).

It was in the early 1990s that researchers first cloned a specific melanoma-derived antigen to induce an immune response by triggering cytotoxic T cells (6, 34, 74). However, it was not until 2010 that the FDA approved the very first autologous cancer vaccine, known as sipuleucel-T, for treatment of castration-resistant prostate cancer (6, 21, 75). This dendritic, cell-based vaccine appeared to extend overall survival of patients during the clinical trials but unfortunately in the clinical setting had no effect on disease progression (6, 75).

Other autologous cancer vaccines are being studied in numerous laboratories all over the world including University of Pennsylvania researchers who are testing an experimental breast cancer vaccine (21, 76). It is widely accepted that HER-2/neu (ErbB) oncogene family plays an important role in growth, development and metastasis of several tumor types including ovarian and breast cancer (77). HER-2/neu (ErbB) was found to be expressed very early in the breast cancer development and the expression of this gene was associated with a significantly increased risk of cancer recurrence after treatment (77). The hope is that the anti-HER-2 response provided by the lab-manipulated Th1 immune cells can be successfully restored by a cancer vaccine (77).

The second type of cancer vaccines, allogenic vaccines, are based on the cells grown in the laboratory; non-self cells (21). This type of vaccine is harder to develop but more appealing because it is potentially less expensive to manufacture (42, 78). The aim is to trigger the immune system instead of attacking a particular cancer cell so this form of treatment has potential against any type of cancer. (42). Despite considerable research effort none has yet been shown to be effective enough for FDA approval (21, 78). One of the very first and most promising clinical trials involved electrofusing allogenic dendritic cells with autologous patient cancer cells from stage IV metastatic renal carcinoma (79). The investigation remains at phase I and II clinical trial stage despite over a decade of intensive research.

All the fore-mentioned cancer vaccines are based on whole cells but there has also been some success in developing cancer vaccines from cancer cell components such as proteins or DNA (21). Those particles might be administered alone or bounded to specialized carriers such as viruses, plasmids or special nanoparticles (4, 21). Therapeutically they may be used alone or as an adjunct with, for example, immune-stimulating molecules (21). There are many on-going clinical trials involving this type of vaccine with targets that include melanoma, breast cancer and prostate cancer (21).

There has also been early success in vaccine clinical trials involving multiple cancer-specific neoantigens that confer high patient-specificity (80, 81). Neoantigens are antigens encoded by mutated genes and present on a tumor surface, therefore since several years they are extensively studied due to their seminal role in cancer immunotherapy (81, 82). As a result of tumor-specific somatic mutations, neoantigens are not present on the surface of normal tissue cells (82, 83). Being highly immunogenic, they can activate CD4+ and CD8+ immune response, providing a perfect target for T cell based cancer immunotherapies (81). Several preclinical studies have already shown the feasibility and efficacy of neoantigen-targeting cancer vaccines in mice models of tumors, for example colon carcinoma, melanoma, glioma and sarcoma (84). Although still in very early stages, approaches combining neoantigens-based therapies with other types of immunotherapy, such as checkpoint inhibitors, as well as conventional treatments, are already ongoing (83, 84).

## CAR T Cells and Adoptive Cell Therapy (ACT)

This form of cancer therapy is a recent breakthrough although the first attempts date back to the 1902 Berlin when Blumenthal and E. von Leyden attempted to vaccinate their patients against cancer using tumor tissue derived from the patients themselves (8, 85). As a vaccine, they used an autologous tumor cell suspension and administered it to several patients with advanced cancer (85). Some subjective improvement was noted but without significant tumor reduction (85).

Adoptive Cell Therapy (ACT) involves the isolation of patient's T cells (recently also termed NK cells), which are tumor-specific, modification and multiplication of those cells in the laboratory and then re-injection back to the patient circulation (4, 86). There are many different ways of modifying cells but one of the most successful is CAR T-cells therapy, sometimes dubbed as a version of ACT. The first use of genetically engineered T cells derived and modified in this way to target cancer cells was reported in 1989 (87, 88). Chimeric antigen T cells (CAR T-cells), were described for the first time in the mid-1990's but failed in preclinical studies or early clinical trials due to the technical intricacies and knowledge gaps that would be remedied only a few years later (89). CAR T-cells therapy was to prove a huge success although it was not without problems as several patients developed a, cytokine storm, a potentially lethal adverse effect if left untreated (1, 4). Nevertheless, relapsed B-cell acute lymphoblastic leukemia in children was the first disease to be FDA approved for CAR T-cells therapy (2017) and this was followed in 2018 by approval for diffuse large B-cell lymphoma and certain other types of lymphoma (4, 90–93).

## Oncolytic Viruses

Oncolytic viral therapy is a revolutionary emerging class of cancer therapeutics that is difficult to classify unambiguously. They are located between immunotherapy and biological therapies of cancer and use existing biological agents to treat cancer. Genetically modified viruses lack their initial virulence but they are still able to penetrate and lyse cancer cells (4). Bursting, dying cancer cells release many molecules that further attract immune system cells, aggravating the immune attack and the overall inflammatory potential of the site (4, 22).

The first oncolytic virus, T-VEC, was approved by the FDA in 2015 for the treatment of metastatic melanoma (4). T-VEC is a herpes simplex 1 virus, genetically modified in a way that allows it to express granulocyte-macrophage colony-stimulating factor (GM-CSF), a powerful cytokine that attracts many types of the immune cells (4, 22). Injections are given directly into the tumor site, especially in metastasis and those regions which cannot be removed surgically. Other oncolytic viruses showing promising results in the clinical trials are Pexa-Vec (against hepatocellular carcinoma), CG0070 (against bladder cancer) and G47Δ (against glioblastoma and prostate cancer), among others (86).

Despite the successful results, there is at least one important disadvantage of using oncolytic viruses: it is the acquired immunity, specific against the virus used. It might effectively disrupt any repeat therapy in the same patient, if used again (86).

## Future Trends

One of the most important challenges in understanding immuno-oncology therapies arise from the complex interactions between a patient's immune system and the cancer's biology. Despite of all, cancers are populations of cells, and all populations are subject to evolutionary forces. Nowadays, there are many emerging trends in immuno-oncology, with checkpoint inhibitors, chimeric antigen T cells (CAR T-cells) and adoptive T-cell therapy (ACT) being the most promising (6). CRISPR/Cas9 gene editing technique has been used to develop CAR T-cells since 2017 (4, 8, 94, 95). At the end of 2018 another breakthrough occurred: the direct reprogramming of mice and human fibroblasts into immune system cells, specifically, antigen-presenting dendritic cells, opening a new line of therapeutic possibilities (96). Our immune system has an amazing capacity for remembering disease-causing antigens so immunotherapy promises a unique opportunity to treat cancer successfully and achieve prolonged remission.

Every cancer cell is estimated to have over 11,000 genomic mutation differences from healthy cells around the tumor (97). Some lead to tumor-associated antigens expressed on the cell's surface and thus become a potential target for new antibody-based therapies. On the other hand, some tumors are known to lose their MHC class I expression, remaining a great immunotherapy challenge (86, 98, 99). Moreover, the entire tumor microenvironment is known to impact cancer growth, development and mediate potential treatment, including the microbiome (100, 101). Several preliminary trials of fecal microbiota transplantations (FMT) have been already conducted with promising results (101, 102).

The identification of relevant biomarkers is a key part of the process. For immune checkpoint inhibitors the level of expression of CTLA-4, PD-1 or PD-L1 genes is measured before drug administration although good outcomes are reported with low level of expression (42). It is also well established that tumors with greater overall mutational load are mostly responsive to checkpoint inhibitor therapy (42). Moreover, cancers with microsatellite instability as a result of mismatch-repair deficiency are known to exhibit particularly strong response to the PD-1 blockade, irrespectively of the cancer type (42). One of the most important challenges in science is to discover why some patients respond to the immunotherapy so perfectly, whereas others are not sensitive to this form of treatment at all. Furthermore, some patients are thought to develop cancer hyperprogression after immunotherapy treatment and the reason for this rare response remains unknown (103).

Immunotherapy in oncology has shown promising responses in a many patients, but acquired resistance could be also a real challenge. The potential mechanisms of relapse include downregulation of tumor antigen presentation, so T cells no longer recognize the tumor cells, loss of T cell function of the host and possible development of escape mutation variants in target cancer cells (104). Clinical studies and the search for new pharmaceuticals are outstripping our current knowledge in cutting-edge immunotherapy and immunooncology. If new, better cancer therapies are to be discovered and existing ones improved there has to be urgent expansion in funding and support for basic science research into the complex and fascinating interplay between the immune system and cancer cells.

## Author Contributions

PD and TD contributed to the design and implementation of the research and to the writing of the manuscript.

### Conflict of Interest

The authors declare that the research was conducted in the absence of any commercial or financial relationships that could be construed as a potential conflict of interest.
